# Current advances in precious metal core–shell catalyst design

**DOI:** 10.1088/1468-6996/15/4/043502

**Published:** 2014-08-05

**Authors:** Xiaohong Wang, Beibei He, Zhiyu Hu, Zhigang Zeng, Sheng Han

**Affiliations:** 1 Department of Chemistry, Shanghai University, Shanghai 200444, People’s Republic of China; 2 New Energy Material Lab, Shanghai Institute of Technology, Shanghai 200435, People’s Republic of China; 3 Institute of NanoMicroEnergy, Shanghai University, Shanghai 200444, People’s Republic of China

**Keywords:** core–shell catalyst, precious metal core, precious metal shell, silica shell, metal oxide shell

## Abstract

Precious metal nanoparticles are commonly used as the main active components of various catalysts. Given their high cost, limited quantity, and easy loss of catalytic activity under severe conditions, precious metals should be used in catalysts at low volumes and be protected from damaging environments. Accordingly, reducing the amount of precious metals without compromising their catalytic performance is difficult, particularly under challenging conditions. As multifunctional materials, core–shell nanoparticles are highly important owing to their wide range of applications in chemistry, physics, biology, and environmental areas. Compared with their single-component counterparts and other composites, core–shell nanoparticles offer a new active interface and a potential synergistic effect between the core and shell, making these materials highly attractive in catalytic application. On one hand, when a precious metal is used as the shell material, the catalytic activity can be greatly improved because of the increased surface area and the closed interfacial interaction between the core and the shell. On the other hand, when a precious metal is applied as the core material, the catalytic stability can be remarkably improved because of the protection conferred by the shell material. Therefore, a reasonable design of the core–shell catalyst for target applications must be developed. We summarize the latest advances in the fabrications, properties, and applications of core–shell nanoparticles in this paper. The current research trends of these core–shell catalysts are also highlighted.

## 1. Introduction

A catalyst is a substance that can change the reaction rate by decreasing the activation energy of a reaction and of itself, but never take part in such reaction. These substances create a convenient surface for the reaction to occur. Precious metals are frequently used as active components of various catalysts. Increasing the catalytic activity and stability of precious metals has always been an important research topic because of the limited quantity of these materials. Previous studies show that the designing of nanoparticles with a core–shell architecture can improve the physical and chemical properties of the material by combining multiple functionalities on a nanoscopic length scale and by providing new active interfaces as well as synergistic effects among different components [1–5]. The development of core–shell particles has been reviewed previously [6–13] and the related materials have also been used broadly in the catalytic area [14–22].

Extensive efforts have been made over the recent years in the designing and the controlled preparation of core–shell structure catalysts with precious metals. These metals can be used both as the core and the shell material according to different purposes.

To preserve these materials, precious metals are mainly used as shell materials of the core–shell structure. The surface area of the precious metal can greatly increase in this structure, which subsequently improves the catalytic activity. A typical example that demonstrates such phenomena is the use of a thin Pt shell in engineered core–shell nanoparticle catalysts, which is among the best solutions to reduce the cost of the catalyst in next**-**generation fuel cell systems [23].

When precious metals are used as core materials, the stability of the catalyst could be improved because of the protection from the shell material. Silica is considered the most popular shell material for many decades because of its simple and robust synthesis from tetraethoxysilane (TEOS) [24]. However, the chemical inertness of silica has made this material an unsuitable catalyst component in some reaction systems. Metal oxides have also been used as shell materials to improve the activity of the core–shell catalyst, but the fabrication of this catalyst remains a challenging task [25].

The core–shell structure can improve the catalytic activity or the stability of the precious metal catalysts as well as provide new functions to the catalyst. Based on their structure, precious metal core–shell catalysts can be mainly classified into core–precious metal shell and precious metal core–shell. In the core–precious metal shell, the precious metal shell plays an active part and the core material takes multiple functions. The core material can offer significant improvements in the activity and selectivity of the catalysts by increasing the surface area of the active part and enhancing the synergistic effects. Using magnetic metal as a core material also facilitates the separation of the catalysts from the reaction mixtures by using a magnet. The core–shell catalysts with a thin precious metal shell or monolayer-based precious metal nanoparticles are of great interest in most cases [26–33]. However, the precise fabrication of this kind of core–shell catalyst remains a great challenge. In the precious metal core–shell, the precious metal core plays an active part and the shell materials play a protective role. The catalytic stability of the precious metal in this catalyst can be improved by confining the transfer of the active particles and by protecting these particles from a damaging environment. Unfortunately, such activity is often degraded because of the coverage of the shell layer. Therefore, maintaining the high activity of this core–shell catalyst remains a problem.

Many approaches for the preparation of core–shell catalysts have been developed recently. These approaches are generally categorized into ‘top**-**down’ and ‘bottom**-**up’ approaches. The former includes laser**-**beam processing and mechanical techniques, whereas the latter includes layer**-**by**-**layer coating, chemical reduction, electrochemical deposition, and sol**-**gel processes. The sol**-**gel method is regarded as the most versatile of these approaches because such method does not require any complicated processes or instruments to synthesize the core–shell catalyst with complex structures. However, this approach still has its own limitations and can only be used for synthesizing certain materials.

We mainly focus on the two abovementioned dominant precious metal core–shell catalysts in this paper. The fabrication methods and the properties of these catalysts are summarized and described in detail. Afterwards, the design and fabrication of the novel core–shell catalysts according to the special requirement are briefly highlighted.

## 2. Core–shell catalysts with precious metal shell

Core–shell catalysts with precious metal shell and various cores have been fabricated for special purposes. As a typical noble metal, platinum (Pt) becomes very important in fundamental studies and industrial applications. Pt was used as the representative metal in this study to interpret the recent advances in the properties, applications, and synthesis of core–shell catalysts with precious metal shell.

**Figure 1.**
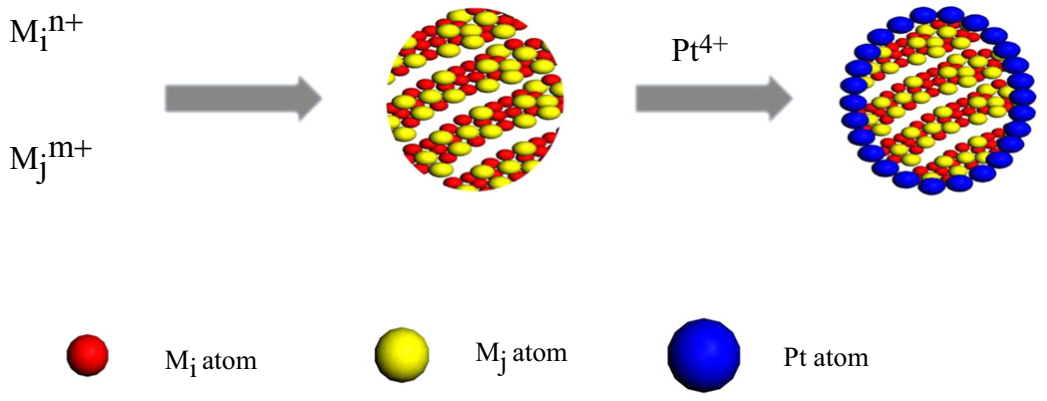
Schematic of the synthetic procedure of the MiMj–Pt core–shell catalyst.

Pt is often used in the form of nanoparticles to create a high surface area for catalysis application. Therefore, Pt is identified as the best catalyst for both the anode and cathode of the proton exchange membrane fuel cell (PEMFC). However, the high cost and instability of Pt prevent PEMFC from having widespread applications. Moreover, the sluggish kinetics at the cathode has a profound effect on the degradation of fuel cell efficiency. Therefore, the improvement of the electrocatalytic activity and durability of a Pt-based catalyst with a low Pt content has been thoroughly investigated. Several strategies have been formulated to address this problem, such as the production of Pt nanostructures with high surface areas and a high catalytic performance as well as the combination of Pt with other materials to improve its performance by modifying the electronic and geometric structures of the surface Pt atoms. In this regard, the synthesis of the core–shell and the core–shell-like catalysts with Pt shell has also attracted significant research interest. Figure 1 illustrates the synthetic procedure of the M_i_M_j_–Pt core–shell structure, where M_i_ and M_j_ represent metallic elements. The illustration shows that the specific surface area of Pt can be greatly improved by combining Pt with other materials. Zhang *et al* [34] prepared the catalyst of Pd–Sn core and Pt shell, which demonstrated a high oxygen reduction reaction (ORR) performance. In principle, many materials can be used as the core. These materials can be mainly classified into three types, namely, precious metal, non-noble metal, and mixture or alloy.

### 2.1. Core–shell catalyst with platinum shell and precious metal core

**Figure 2.**
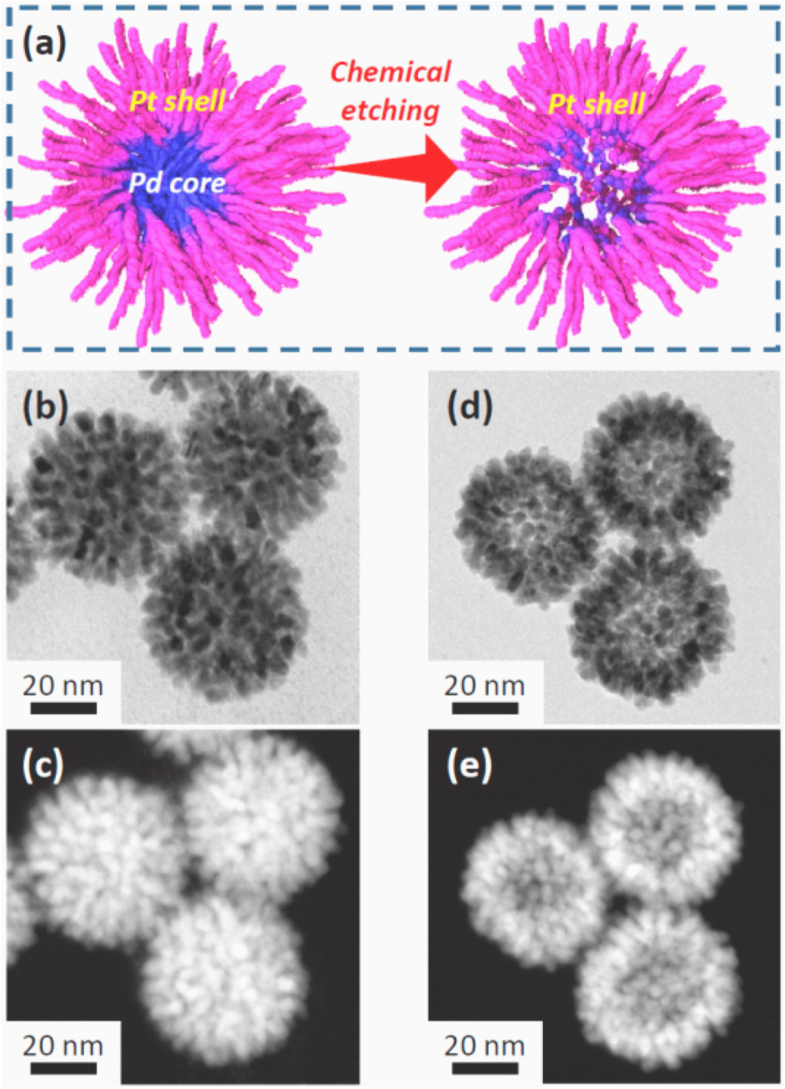
(a) Schematic of the formation of a bimetallic dendritic nanocage with a hollow interior and a porous dendritic wall. (b)–(e) Transmission electron microscopy (TEM) and high-angle annular dark-field scanning TEM (HAADF-STEM) images of dendritic Pt-on-Pd nanoparticles. (b)–(c) Before chemical etching; (d) and (e) after chemical etching [38].

Metal–metal, such as precious metal–platinum core–shell nanoparticles, are considered important catalysts because a metal nanoparticles shell, especially mesoporous shell, provides many catalytically active sites and a high accessibility of guest to the core surface for catalytic reaction [35, 36]. The core–shell structure can also generate new catalytic properties that are driven by the electronic interactions between the Pt shell and the second core metal [36, 37]. Therefore, the synthesis of the Pt-based core–shell catalyst is of great significance. As shown in figure 2 [38], the Pd–Pt core–shell nanoparticles with dendritic shell can be synthesized via a sophisticated chemical etching approach. These nanoparticles possess a superior catalytic activity for methanol oxidation reaction as compared to other Pt catalysts. Other metals, such as Au and Ag, can also be used as sacrificial templates for the preparation of hollow Pt nanoparticles or core–shell particles [36, 39].

Table 1 lists the applications, performance advantages, defects, and catalytic activities of typical core–shell catalysts with platinum shell and precious metal core. The prices of some precious metals are also listed on this table. Among these metals, Pt is the most expensive, Au and Rh are cheaper, and Ru is the cheapest. Given that Pd, Ru, and Rh are all VIIIB group elements, which properties are analogous with those of Pt, they may be considered as highly suitable replacements for Pt. However, given the expensiveness of Rh, Rh–Pt fails to attract much interest as a research topic. Recent reports suggest the Pd–Pt bimetallic catalyst, especially the Pd–Pt core–shell catalyst, as a promising replacement for the Pt catalyst because of its unique characteristics, which are not merely combined from the properties of the two constituent metals [40–49]. Table 1 shows that the methanol electron**–**oxidation reaction (MOR) activity on Pd–Pt can be greatly improved. The peak specific area and mass current density for MOR on the Pd–Pt catalyst are approximately 50 mA cm^–2^ and 376 mA mg^–1^, respectively, which are ten times greater than those on the pure Pt catalyst. At the same time, Ru–Pt [22, 50–54], Rh–Pt [55], Au–Pt [56–77], and Ag–Pt [36] also possess a superior catalytic performance in terms of CO tolerance.

**Table 1.** The applications, advantages, defects and catalytic activities for the fuel cell of typical precious metal–Pt core–shell catalysts.

Catalysts (core– shell)	Applications	Advantages	Defects	Peak specific area current density (mA•cm^−2^)	Peak specific mass current density (mA•mg^−1^ Pt)	Prices (US dollars per troy ounce)
Pure Pt	Fuel cell	An ideal cathode and anode catalyst Facile synthesis	High price Poor kinetics of the anode reaction Low tolerance to CO poisoning	∼5 (MOR) (C_methanol_ = 0.5 M) [48] ∼0.15 (ORR) [54]	∼200 (ORR) [54]	Pt: 1496
Pd–Pt	Fuel cell; Hydrogen Storage	Less expensive High ORR activity High MOR activity Superior CO tolerance	Complicated synthesis	∼50 (MOR) (C_methanol_ = 0.5 M) [48]	∼376 (MOR) (C_methanol_ = 0.5 M) [48]	Pd: 737
Ru–Pt	Fuel cell	Low price Superior CO tolerance	Complicated synthesis	∼0.65 (ORR) [54]	∼950 (ORR) [54]	Ru: 80
Au–Pt	Fuel cell; Photo electrochemical solar cell	Less expensive Superior CO tolerance Superior durability for oxygen reduction High activity for formic acid oxidation	Complicated synthesis	∼20 (MOR) (C_methanol_ = 1 M) [59]	∼632 (MOR) (C_methanol_ = 1 M) [59]	Au:1336
Rh–Pt	Preferential CO oxidation (PROX)	Less expensive High PROX selectivity	Complicated synthesis			Rh: 1010

In summary, the core–shell catalysts prepared by depositing Pt on other precious metals have attracted significant research interest because the Pt shells supported on such cores demonstrate a catalytic activity and durability that are remarkably greater as compared to those of the pure Pt catalyst; such enhancement is a result of the modification of the electronic and geometric structures of the surface Pt atoms [19, 20, 23, 78, 79, 80–82].

### 2.2. Core–shell catalyst with platinum shell and non-precious metal core

Although precious metal–Pt core–shell catalyst is a fine replacement for the Pt catalyst, this material is extremely expensive. Recent studies show that the Pt electrocatalyst can achieve a high catalytic activity and utilization efficiency by replacing the core of the nanoparticles of the precious metal electrocatalysts with low-cost or non-precious metals or by reproducing a nanosphere with high surface-to-volume ratios. Theoretical and experimental studies show that arranging Pt metals as thin shells on the proper non-noble metal (e.g., Fe, Co, Ni, Cu, Cr, and V) cores not only reduces their usage but also significantly enhances their catalytic activity [83–85]. The atomic neighborhood of two dissimilar metals can modify the monometallic behavior and generate the desired new properties because of the short-range electronic charge transfer (ligand effects [86–90]) and the altered lattice constants (geometric effects [89, 91]).

Table 2 shows the applications, performance advantages, defects, and catalytic activities of typical core–shell catalysts with platinum shell and non-noble metal core. Given that Fe, Co, and Ni are VIIIB group elements that possess properties that are analogous with those of Pt, they are deemed suitable candidates to replace Pt as the core material. The mass activity of these core–shell catalysts toward ORR has been improved 4–22 times, which has subsequently decreased the cost of the catalyst [92–95]. Furthermore, the magnetism of these elements can also facilitate the recovery of the catalysts. Aside from the abovementioned non-noble metals, Cu is also a favorable candidate for improving the catalytic activity and decreasing the cost of the Pt catalyst because Cu is a member of IB group elements that possess properties that are similar with those of Au and Ag.

**Table 2.** The applications, advantages, defects and catalytic activities for the fuel cell of typical non-noble metal–Pt core–shell catalysts.

Catalysts (Core– shell)	Applications	Advantages	Defects	Peak specific area current density (mA•cm^-2^)	Peak specific mass current density (mA•mg^-1^ Pt)
Pure Pt	Fuel cell	An ideal cathode and anode catalyst Facile synthesis	High price Poor kinetics of the anode reaction Lower tolerance to CO poisoning	∼5 (ORR) [93]	∼20 (ORR) [93]
Fe–Pt	Fuel cell	Low price Easy recovery due to the magnetism Superior activity to ORR	Complicated synthesis		~100 (MOR) [94]
Co–Pt	Fuel cell	Low price Easy recovery due to the magnetism Superior activity to ORR	Complicated synthesis	~6 (ORR) [95]	~465(ORR) [95]
Ni–Pt	Fuel cell	Low price Easy recovery due to the magnetism Superior activity to ORR	Complicated synthesis	~3 (ORR) [92]	
Cu–Pt	Fuel cell	Inexpensive Simple galvanic-replacement reaction with Pt precursors, which can generate nanoporous core–shell structure Enhanced catalytic activity and stability		~5 (ORR) [93]	~80 (ORR) [93]

Other metals, such as Cr, V, and Sn, have also been used as core materials to fabricate non-noble metal–Pt core–shell catalysts. Chen *et al* [96, 97] synthesized the Sn–Pt core–shell nanospheres that were used for electrochemical catalysts. Given their low electrical resistivity, low melting point, and high surface tension, the structure of these catalysts can be easily tuned.

### 2.3. Core–shell catalyst with platinum shell and bi-metal core

The properties of the core–shell catalyst with platinum shell can be tuned by changing the composition of the core materials. Given that metals demonstrate different advantages, the catalytic performance of the core–shell catalyst may be improved by combining one metal with another to serve as the core. Therefore, a core–shell catalyst with platinum shell and bi-metal core has been fabricated. Pd–M (precious metal or non-precious metal)–Pt has attracted much research interest as the Pd–Pt core–shell catalyst is a promising replacement for the Pt catalyst. Wang *et al* [98] investigated the ORR activity of Pt, Pd–Pt, and Pd–Co–Pt core–shell nanoparticles and found that the Pt-monolayer catalyst on the 4 nm Pd and 4.6 nm Pd_3_Co cores exhibited 1.0 mA mg^–1^ and 1.6 mA mg^–1^ Pt mass activities, respectively, which were about five and nine times greater than those of 3 nm Pt nanoparticles. The Pd–Sn–Pt catalyst has also been synthesized by Zhang *et al* [34] to reproduce a nanosphere shell with high surface-to-volume ratios. Pd–Sn is considered a favorable core for the following reasons: (1) Pd is a fine type of electrocatalyst that can be used under acid conditions and has a surface that favors the reductive deposition of Pt [34, 99]; and (2) Sn is an active metal that can contribute to decreasing the cost of catalysts and can be easily replaced by Pt [34]. For the same purpose, Hui *et al* [100] proposed a general strategy to prepare carbon-supported Pd–Cu–Pt nanoparticles with an intimate contact between Pt and Pd–Cu. The MOR and ORR catalytic activities of the catalyst is higher than those of the Pt/C. Yang *et al* [101] prepared Pd–Ag–Pt core–shell catalyst that exhibited a superior catalytic activity toward the ORR in fuel cells. The Ag component is crucial for constructing the multiple**-**twinned structure of the core–shell nanoparticles, whereas the Pd component is used to reduce the tensile strain effect of the Ag on the deposited Pt layer, which renders the Pd binding energy in core–shell Pd–Ag–Pt nanoparticles. Aside from the above examples, the Pd–Pt–Pt, Mo–Pt–Pt, and Pb–Pt–Pt core–shell catalysts have also been prepared to improve the performance of the Pt-based catalyst [102–105]. It is obvious that, the core material composition is a key factor that affects catalytic activity. Choosing and tuning the composition of the core material may be another interesting research topic.

### 2.4. The fabrication of the core–shell catalyst with platinum shell

In summary, the activity and stability of the Pt-based electrocatalyst can both be improved by the fabrication of the core–shell structure. Numerous methods have been developed for the preparation of core–shell catalysts with Pt shell.

Table 3 shows the basic operations and features of different fabrication methods. The fabrication of the core–shell catalyst with Pt shell follows three methods mainly. The general method is the wet chemical method, which includes the one**-**step and two**-**step (sequential reduction) methods [48, 66, 69, 73, 75]. These methods adopt many techniques (such as microwave heating [48] and ultraviolet irradiation [75]) and sometimes use surfactant as a template for modifying the surface morphology of the material. The advantages of this method, especially the one**-**step method, lie in their simple operation and gentle operation condition.

**Table 3.** Fabrication methods for the Pt-based core–shell catalysts.

Fabrication methods	Examples	Basic operations	Features
Wet chemical synthesis methods	One-step microwave heating method [48]	Heating the mixture of metal precursors, surfactants, and reductants in the microwave synthesis system	Simple and fast
	One-pot simultaneous reduction method [73]	Mixing Nafion with deoxidized Milli-Q water for 30 min Adding metal precursors under an atmosphere of nitrogen in the dark Adding reductants and stir for 1 h at 0 °C	Simple and has a gentle operation condition
	One-step ultraviolet irradiation method [75]	Mixing metal precursors with plolyethylene glycol and acetone and then exposing the mixture to ultraviolet irradiation	Simple, fast and has a gentle operation condition
	Successive reduction method [66, 69]	Reducing one metal precursors to form the nanoparticles Using the nanoparticles as seeds and reducing the other metal precursors to form the core–shell structure	Multiple steps, high reaction temperature, and long duration
Electrochemical synthesis methods	Electrochemical self-organization method [106]	Treating the substrate Electrodepositing precious metal on the substrate	Conductive substrates and an electrochemical cell are needed
Galvanic displacement method [23, 49, 107, 108]	Displacing under potentially deposited (UPD) Cu atoms on a substrate metal surface by another metal monolayer	Conductive substrates and an electrochemical cell are needed
Atomic layer deposition methods	Atomic layer deposition method [109]		A viscous flow reactor and a rigorous operation condition are needed

Electrochemical synthesis is another important method for fabricating the Pt shell catalyst. This method includes the general electrochemical method [106] and the Galvanic displacement method [23, 49, 107, 108]. Electrochemical synthesis requires conductive substrates and an electrochemical cell, and its process is more complicated than that of the wet chemical method.

Atomic layer deposition can also be used to synthesize the Pt shell catalyst [109]. This technique has promising uses for the production of uniform precious metal nanoparticles on high surface area supports because of its sequential and self**-**limiting surface reactions. However, this method has a high instrument requirement and a severe operation condition.

## 3. Core–shell catalyst with precious metal core

Supported precious metal catalysts have been extensively studied because of their unique properties that greatly enhance their catalytic activity for various reactions. The main function of the catalyst support is to offer an effective surface and a suitable porous structure. The interaction between the active component and the support also plays an important role in the catalytic reaction. However, the aggregation and poisoning of metal nanoparticles will minimize their surface area and surface energy, which results in a loss of nanostructure**-**specific properties. The encapsulation of precious metal nanoparticles as a core inside of a supported material as a shell has recently attracted much research attention. This core–shell structure can maintain the size and shape of the precious metal nanoparticles as well as protect them from damage. The stability and compatibility of the particles can be enhanced by encapsulating nano precious metal particles in a stable shell. At the same time, the electron charge, reactivity, and functionality of the enwrapped materials can also be changed in the process [110–112]. Several materials can be used as shell materials, such as mesoporous silica, metal oxides, and other porous materials. Mesoporous silica and metal oxides, which are considered the general catalyst supports, are also identified as the most important among these materials.

### 3.1. Core–shell catalyst with precious metal core and mesoporous silica shell

Ordered mesoporous silica materials were fist synthesized in 1992 [113] and the interest has been extended from the mesoporous silica nanoparticles [114] to the thin films [115–118]. The progress in this area has been reviewed [119–121]. Since its discovery, mesoporous silica has been extensively used as a catalyst support because of its high thermal stability, controllable morphology, and desirable surface area. Silica particles also play an important role in the preparation of core–shell nanoparticle systems because of their easy functionalization and simple synthesis from the TEOS monomer. Liz-Marzán *et al* [122] were the first to report the synthesis of gold–silica core–shell particles using the Stöber method. However, given that the silica synthesized by the classical Stöber method is usually non-porous, this property restricts the application of the core–silica shell in the catalytic area. This problem can be effectively resolved by preparing mesoporous silica (mSiO_2_) [123–125]. The first core–mSiO_2_ structure that was formed by the Stöber process was reported by Büchel *et al* [126], which was followed by an increasing attention from many researchers, such as Somorjai *et al* [24] and Zhao *et al* [127–131]. The core material has been extended to all kinds of materials, such as metals and oxides. Numerous studies have been conducted to prepare various precious metal core–silica shell nanostructures for a wide range of applications. The categories, features, applications, and fabrications of the core–shell catalysts with precious metal core and silica shell are summarized in table 4 [16, 24, 112, 132–140, 141–144, 145–150, 151].

**Table 4.** Features of the core–shell catalysts with precious metal core and silica shell.

Catalysts (Core–shell)	Examples	Applications	Features	Fabrication methods and operations
Single precious metal–silica	Pt–silica [24, 139, 141, 149] Au–silica [133, 135–138, 140, 143–146, 150, 151] Ru–silica [112] Ni–silica [112]	CO oxidation Water–gas shift reaction Ammonia decomposition Electrocatalyst	Easy to fabricate More active and stable than silica-supported catalysts Not as active as metal oxide-suppoted catalysts	Colloidal methods: Precious metal nanoparticles preparation by reduction method Coating of nanoparticles by TEOS hydrolyzing Calcination
Precious metal composites –silica	FeO_x_–Au–silica [132, 134] Pd–SiO_2_–silica [16, 142]	Reduction of o-nitroaniline to benzenediamine One-pot oxidation involving the synthesis of H_2_O_2_	Easy to fabricate High activity and selectivity High stability	Colloidal methods: Synthesis of oxides Synthesis of precious metal composites Coating of precious metal composites with silica
Precious metal alloy or bi-metal nanoparticles–silica	Au–Pd–silica, Au–Pt–silica, Au–Ag–silica [147, 148]	Suzuki cross-coupling reaction	Easy to fabricate High catalytic activity and selectivity	One-pot hydrothermal reaction method: Preparation of silica spheres Introducing Au–Pd alloy *in situ* by the hydrothermal reaction of the salts

**Figure 3.**
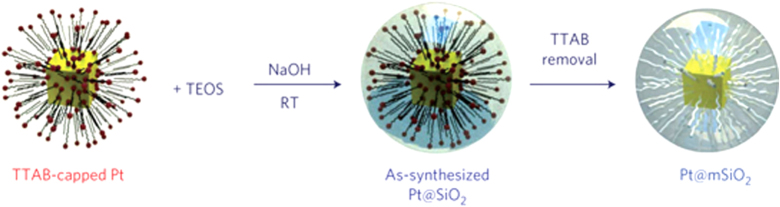
Schematic of the synthesis of Pt–mSiO_2_ nanoparticles. Pt nanoparticles were synthesized using the tetradecyltrimethyl ammonium bromide (TTAB) surfactant as the capping agent, and used as the core particles. Second, as-synthesized Pt–SiO_2_ particles were prepared by polymerizing TEOS around the TTAB [24]. RT stands for room temperature.

**Figure 4.**
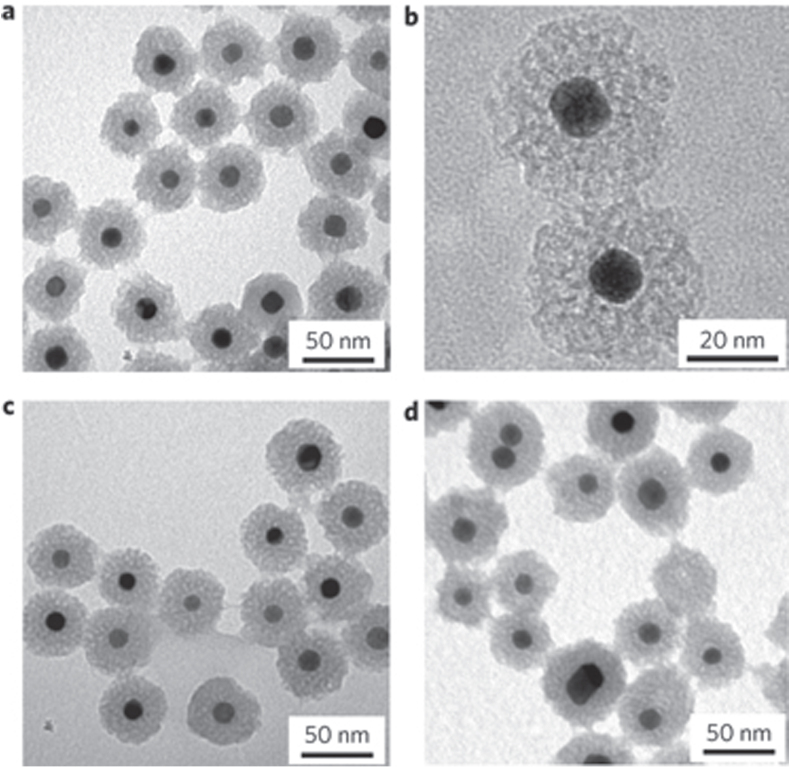
Thermal stability of Pt–mSiO_2_ nanoparticles. (a)–(d) TEM images of Pt–mSiO_2_ nanoparticles after calcination at 350 °C (a) and (b), 550 °C (c) and 750 °C (d) [24].

As shown in table 4, the core–shell material with precious metal core and silica shell can be used as the catalyst of many reduction or oxidation reactions. This material has an easy fabrication process, high activity, high stability, and is generally fabricated using colloid methods, which involve three operation steps (table 4). The formation mechanism of the precious metal–mSiO_2_ core–shell structured nanoparticles has been depicted in detail by Somorjai *et al* [24] (figure 3), who also reported the synthesis of the thermally stable Pt–mSiO_2_ core–shell nanocatalyst for a high**-**temperature reaction. The TEM image of the material that is prepared by the above method is shown in figure 4 [24]. This material can maintain its core–shell configuration up to 750 °C, and the ignition temperature of CO on the catalyst is about 300 °C. Although the catalytic activity of the precious metal–silica core–shell catalyst is higher than that of the general silica-supported precious metal catalyst, such activity remains very low because of the inertness of silica. Therefore, replacing silica with other active materials, such as metal oxide, may present a favorable alternative.

### 3.2. Core–shell catalyst with precious metal core and metal oxide shell

The precious metal nanoparticles that are encapsulated in metal oxide are extremely active and stable in many reactions because a group of ‘active’ support materials, such as Fe_2_O_3_, TiO_2_, SnO_2_, NiO_x_, CoO_x_, MnO_x_, CeO_x_, and CuO_x,_ show a higher catalytic activity than that of ‘inert’ materials, such as SiO_2_, when combined with precious metal nanoparticles [152]. These ‘active’ reducible transition metal oxides serve as an oxygen supply during reactions. A synergistic mechanism may occur at the precious metal–metal oxide interface, and with the metal oxide being part of the catalytic process, the electrons are transferred from the oxide support to the precious metal nanoparticles [153]. Table 5 shows the features, applications, and fabrications of typical core–shell catalysts with precious metal core and metal oxide shell.

**Table 5.** Features of typical core–shell catalysts with precious metal core and metal oxide shell.

Catalysts (Core–shell)	Examples	Applications	Features	Fabrication methods and operations
M–TiO_2_	Pt–TiO_2_ Au–TiO_2_ Pd–TiO_2_ [169–175]	Photocatalytic reaction CO oxidation Water–gas shift reaction	Tunable photoreactivity Controllable chemical and colloidal stability High redox capability	Hydrothermal reaction method: Precious metal nanoparticles preparation by reduction method Coating of nanoparticles by the precursors of hydrothermal reaction Calcination
M–Fe_2_O_3_	Ag–Fe_2_O_3_ Au–Fe_2_O_3_ [178, 179]	Oxidation of VOC CO oxidation	High catalytic performance High stability	Deposition precipitation method: Precious metal nanoparticles preparation by reduction method Coating of precious metals using the homogeneous deposition precipitation of Fe precursors
M–CeO_2_	Pt–CeO_2_ Au–CeO_2_ Pd–CeO_2_ Ag–CeO_2_ [25, 180–184]	Water–gas shift reaction Chemoselective reduction	High catalytic activity and selectivity Simple synthesis method	Microemulsion method: Precious metal nanopaticles preparation by reducing the precursors in microemulsion The oxidation of Ce^3+^ with H_2_O_2_ to form CeO_2_
M–SnO_2_	Au–SnO_2_ [153, 185–187]	Gas sensors	High temperature stability Fair photocatalytic activity	Microwave hydrothermal method: Precious metal nanoparticles preparation by reduction method Coating of precious metals in a microwave oven
M–Cu_2_O	Au–Cu_2_O [188–191]	Photocatalysts Electrocatalysts	Precise positional and morphological controllability	Au nanocrystal-directed growth method Au nanoparticles preparation by reduction method Cu_2_O growth on Au nanoparticles

**Figure 5.**
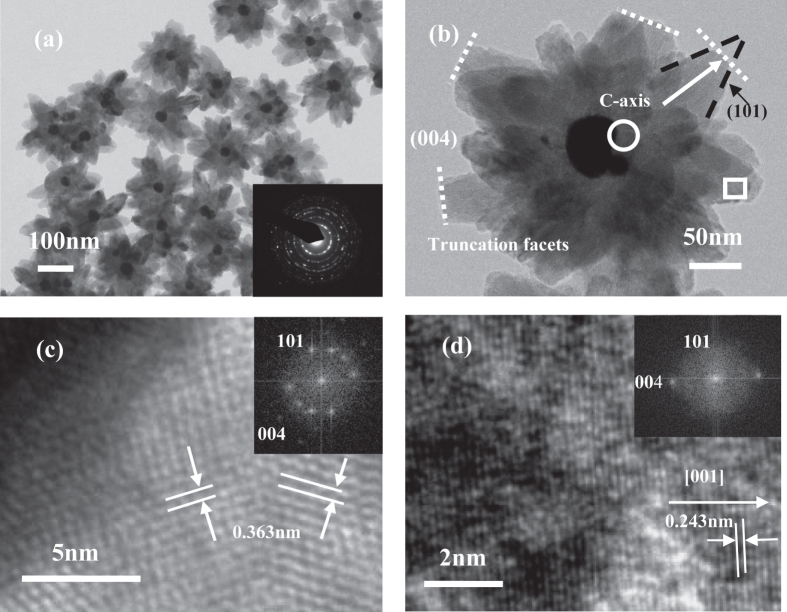
TEM images of the as-prepared core–shell Au–TiO_2_ nanoparticles (a) and individual particle image (b). The inset in (a) is the electronic diffraction (ED) pattern of the individual particle. Local HRTEM images of individual TiO_2_ antenna: root region (c) and external edge of individual antenna (d); the insets in(c) and (d) are their corresponding FFT patterns [174].

TiO_2_ semiconductor has been regarded as one of the most popular photocatalysts, especially in the cleaning of water and the removal of volatile organic compounds (VOC) in air [154–159]. This material has also been used extensively as the support of CO oxidation or water–gas shift reaction catalysts [160–165]. Several studies prove that, in metal–TiO_2_ core–shell particles, the high Fermi energy level of metal cores can efficiently separate the photoexcited electron-hole pairs of the TiO_2_ conductor, which heightens the redox capability of the metal core [166–168]. Table 5 shows that Pt, Au, and Pd–TiO_2_ core–shell catalysts have all been prepared and used for photocatalytic reactions, CO oxidation, and water–gas shift reactions [169–175]. Although these catalysts are very active and stable, the fabrication of metal–TiO_2_ structures remains a great challenge. The hydrothermal reaction method remains as the general method and only few successful examples have been reported to date [174, 176]. Figure 5 shows the flower-like Au–TiO_2_ nanoparticles with shell that are built by packed TiO_2_ nanoparticles [174].

As shown in figures 4 and 5, the main difference between the silica shell and metal oxide shell catalysts lies in their particle size and morphology, which can be ascribed to the fact that the two systems follow different particle growth theories. The large precious metal particles and thick shells of metal–TiO_2_ may lead to a low catalytic activity. However, the flower-like morphology is more suitable than the spherical morphology as a catalyst. Therefore, combining the advantages of the two systems may present another topic that is worth investigating. Lekeufack *et al* [177] obtained different morphologies of the particles by adjusting the proportion of the TiO_2_ precursor in the silica precursor and by displacing the gold particles from the center to eccentric positions, which would generate acron- and raspberry-like structures.

Fe_2_O_3_ serves as a favorable catalyst support because of its fair oxidation and reduction properties [178, 179]. Yin *et al* [179] prepared Au–Fe_2_O_3_ core–shell structures on silica supports and investigated their catalytic activity. The Fe_2_O_3_ shell has a thickness of about 2 nm, and the Au–Fe_2_O_3_ interface is active for CO oxidation. The catalytic activity of silica-supported Au–Fe_2_O_3_ core–shell structure catalyst is greater than that of the Fe_2_O_3_-supported Au catalyst with a comparable gold loading. The silica-supported Au–Fe_2_O_3_ catalysts are stable in CO oxidation at room temperature after 20 h on stream.

CeO_2_ has been identified as an important component of catalysts for many reactions, such as water–gas shift reaction and automotive off-gas purification. This material can store and release both oxygen and hydrogen by forming a surface, bulk vacancies, or an inter-metallic M–Ce compound. Ceria can also serve as a stabilizer for metal and alumina supports by maintaining a high dispersion of the catalytic metals. Table 5 shows that Pt, Au, Pd, and Ag can all be encapsulated into CeO_2_ to form the core–shell structure [25, 180–184]. Mitsudome *et al* [181] successfully synthesized a core–shell Ag–CeO_2_ nanocomposite that comprised Ag nanoparticles with a 10 nm diameter in the core as well as assembled a spherical CeO_2_ with a 3 nm diameter in the shell. This catalyst is easily separable and reusable, with retention of its high catalytic performance.

Sn belongs to the same IVA group as Si, and support an easy fabrication of M–SnO_2_ core–shell structures. In addition, the catalytic activity of SnO_2_ is much higher than that of SiO_2_. Therefore, the M–SnO_2_ core–shell catalyst presents a very interesting phenomenon [153, 185, 186, 187]. Yu *et al* [153] prepared Au–SnO_2_ catalyst for CO oxidation and found that its catalytic activity and stability was much higher than that of the non-encapsulated Au–SnO_2_.

**Figure 6.**
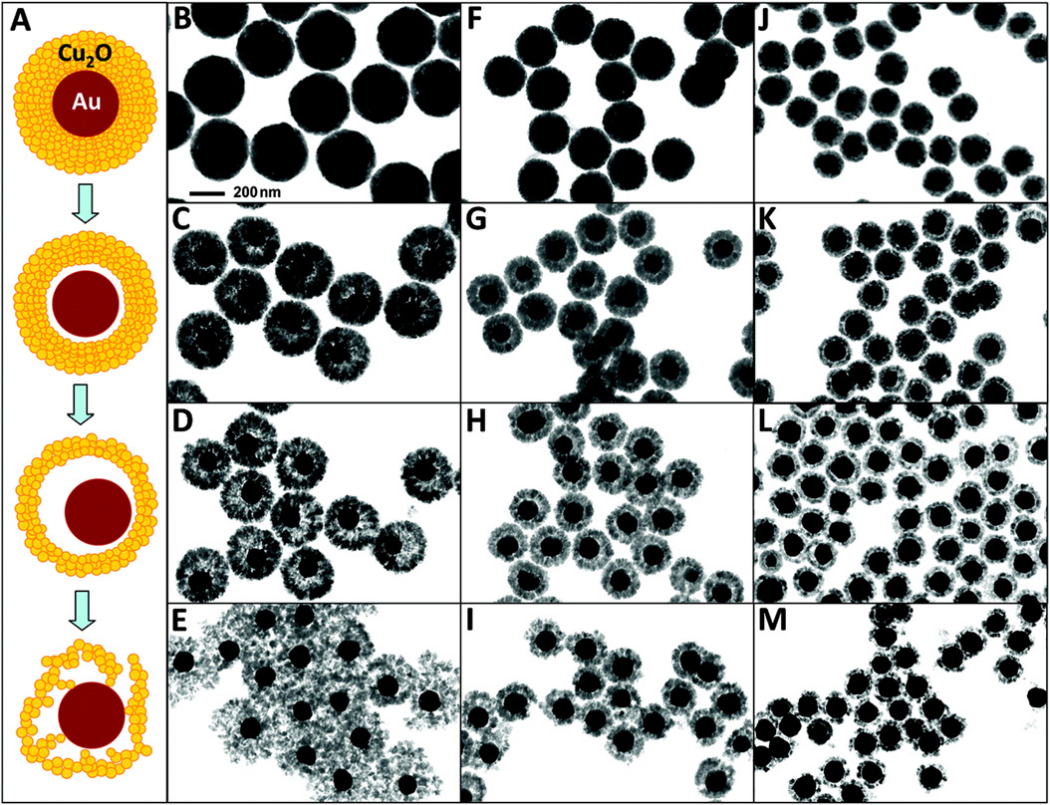
Formation of rattle-like Au–Cu_2_O yolk–shell nanoparticles. (A) Schematic illustration of the structural evolution of Au–Cu_2_O core–shell nanoparticles during the hollowing of Cu_2_O shell. (B)–(M) Bright-field TEM images showing the structural evolution of Au–Cu_2_O core–shell particles with three different outer radii: particles with an average outer radius of 183 nm obtained at (B) 5, (C) 30, (D) 60, and (E) 90 min; particles with an average outer radius of 130 nm obtained at (F) 5, (G) 20, (H) 40, and (I) 60 min; particles with an average outer radius of 98 nm obtained at (J) 5, (K) 20, (L) 40, and (M) 60 min. The scale of all TEM images is the same as in B [191].

Cu_2_O can serve as a photocatalyst or redox catalyst owing to its optical and chemical properties. Therefore, Au–Cu_2_O becomes a favorable catalyst [188–191]. To date, the Au–Cu_2_O core–shell particle remains the only particle with a perfect core–shell structure that is comparable with that of M–SiO_2_ (figure 6) [191].

Overall, the morphology of the core–shell catalyst with precious metal core and metal oxide shell is often irregular. The activity and stability of this catalyst are much higher. Nevertheless, this catalyst has a complicated preparation process and a lower yield. Therefore, a convenient method for increasing the yield of the core–shell catalyst must be developed.

## 4. Problems and challenges

Although the study of precious metal core–shell catalysts has only started a few years ago, they have exhibited a great potential for application in heterogeneous catalysis area owing to their high activity, high stability, high selectivity and other functions. Despite the great advancements in this field, several problems remain unsolvable, which are listed as follows: (1)The efficient fabrication methods of the core–shell structural catalyst are still limited, and each method can only be used to prepare certain materials. (2)The core size and the shell thickness remains difficult to control precisely. (3)The yield of the core–shell catalyst remains low. (4)When a precious metal core is used as the active part of the catalyst, its catalytic activity is always degraded by the shell-block effect. These facts have restricted the application of the core–shell structure material and meanwhile it offers a lot of room for the extension of the fabrication of core–shell catalyst.

## 5. Conclusions

Studies on the core–shell structure catalyst are often conducted to reduce the cost of such catalyst by improving the performance of precious metals. The efficiency of precious metals may be improved by fabricating the precious metal shell as thin as possible, decreasing the size of the precious metal core nanoparticles, and dispersing the core–shell structure catalysts on the support. Therefore, future studies on core–shell structure fabrication must consider controlling the core size and tuning the shell thickness. An increase in the shell pores can also improve the catalytic activity of the precious metal core. These goals can only be achieved by developing new methods or optimizing the technology of the existing methods. Moreover, using the mixture of the easy shaping and chemically active materials (such as silica and metal oxide) as a shell material may also contribute to the fabrication of a high-performance core–shell catalyst that has the combined advantages of different materials.
